# Characterizing the Key Agents in a Disease-Suppressed Soil Managed by Reductive Soil Disinfestation

**DOI:** 10.1128/AEM.02992-18

**Published:** 2019-03-22

**Authors:** Liangliang Liu, Xinqi Huang, Jun Zhao, Jinbo Zhang, Zucong Cai

**Affiliations:** a School of Geography Science, Nanjing Normal University, Nanjing, China; b Jiangsu Center for Collaborative Innovation in Geographical Information Resource Development and Application, Nanjing, China; c Key Laboratory of Virtual Geographical Environment (VGE), Ministry of Education, Nanjing Normal University, Nanjing, China; d State Key Laboratory Cultivation Base of Geographical Environment Evolution, Nanjing, China; e Jiangsu Provincial Key Laboratory of Materials Cycling and Pollution Control, Nanjing Normal University, Nanjing, China; University of Tokyo

**Keywords:** soilborne disease, disease-suppressive agents, environment-microbe interactions, microbial community, reductive soil disinfestation

## Abstract

Many agricultural soil management strategies have been shown to be effective in preventing soilborne diseases. However, their underlying mechanisms of action remain unknown. In this study, we used reductive soil disinfestation (RSD), also named anaerobic soil disinfestation (ASD) and biological soil disinfestation (BSD), as a representative method for disease management and cucumber damping-off diseased soil as a model system to identify the disease-suppressive agents in artificially managed soil. The results showed that RSD created a soil environment that was different from that of the diseased soil, where the pH level and the carbon content were greater. Heat treatment and pathogen or soil microbiota self- and cross-reinoculations resulted in the expansion of various soil microbial communities harbored by the two soil environments, as well as various disease incidences. Environmental factors were the primary determinant of the reassembled bacterial community, followed by initial microbiota, whereas initial microbiota was the key driver of the reassembled fungal community. The relative abundances of the bacterial order *Sphingobacteriales* and fungal order Sordariales, as well as their affiliated genera *Sphingobacterium*, unclassified genus within *Sphingobacteriaceae*, *Zopfiella*, and unclassified genera within Lasiosphaeriaceae and Chaetomiaceae, were negatively correlated with disease incidence and positively associated with RSD-conditioned soil environment. Furthermore, we validated that both the microbial disease-suppressive agent and its adapted abiotic environment contributed to disease suppression. Our results elucidate the abiotic and biotic foundations of soilborne disease suppression under artificial management and highlight that the abiotic environment is as important as the microbial agents in disease suppression.

**IMPORTANCE** Most defined systems have identified microbial elements as the primary factors determining disease suppression, but the involvement of the soil abiotic environment is less defined. The significance of this work is that the soil abiotic environment plays a critical role in the establishment of the soil microbial community and key microbial agents that directly contribute to the prevention of soilborne diseases. We highlight the importance of the soil abiotic environment in disease suppression. Furthermore, we provide a framework for the characterization of disease-suppressing agents in artificially managed soil. These results will gradually close the gap in knowledge on soil environment-microbe interactions.

## Abstract (precis)

Most defined systems have identified microbial elements as the primary factors determining disease suppression, but the involvement of the soil abiotic environment is less defined. The significance of this work is that the soil abiotic environment plays a critical role in the establishment of the soil microbial community and key microbial agents that directly contribute to the prevention of soilborne diseases. We highlight the importance of the soil abiotic environment in disease suppression. Furthermore, we provide a framework for the characterization of disease-suppressing agents in artificially managed soil. These results will gradually close the gap in knowledge on soil environment-microbe interactions.

## INTRODUCTION

Intensive cropping systems are characterized by continuous monocultures or limited crop rotations that often cause soil degradation, involving soil acidification, salinization, the unbalanced supply of nutrients, and the accumulation of soilborne pathogens (1). Particularly, plant diseases primarily caused by soilborne pathogens, such as damping-off and Fusarium wilt caused by Rhizoctonia solani and Fusarium oxysporum, respectively, often lead to economic losses in many important crops (2, 3). In order to reduce the inoculum level of soilborne pathogens, chemical fumigation is largely used in practice. However, the use of several effective chemical pesticides has been restricted, due to concerns of environmental pollution and food safety. For example, the use of methyl bromide, which was once a highly effective soil fumigant, was banned under the Montreal protocol in 2004, largely because of its ability to deplete ozone (4, 5).

In addition, the abundance of soilborne pathogens is not always the sole determinant for soilborne diseases (6), because studies have shown that the physicochemical properties of soil, such as the pH and the contents of nitrogen and organic carbon, significantly influence the development of soilborne diseases (7, 8). However, the physicochemical properties that influence disease suppression either directly or indirectly through their impact on soil microorganisms are still unclear. Furthermore, specific disease-suppressive soils, in which the presence of pathogens cannot result in disease due to the presence of an individual or representative group of antagonistic microorganisms, have been receiving attention for more than a century (9, 10). Studies have identified the underlying mechanisms of disease suppression in these soils and provided new insights on potential control strategies for soilborne diseases (11, 12). However, the establishment of naturally occurring disease suppression in soil is a slow process and can take several years, during which time the disease incidence is often high (9–12) and results in poor acceptance by farmers, especially in China. Thus, the improvement of the ability of soil to suppress diseases through artificial management strategies is the mainstream practice.

Many studies have described interesting soil management approaches, such as organic amendment, that can support plant health, possibly by changing both abiotic and biotic properties, although the underlying relationships between these properties and soil disease suppression remain unclear (13, 14). For instance, reductive soil disinfestation (RSD), also called anaerobic soil disinfestation (ASD) and biological soil disinfestation (BSD), a pre-plant soil disinfestation method that involves the incorporation of organic matter, irrigation to maximum field capacity, and covering of the soil surface with plastic film (15), is summarized in an upland-paddy rotation system that is tolerant to soilborne diseases (16). During RSD treatment, the production of antagonistic compounds, such as organic acids, manganese (Mn^2+^) and ferrous (Fe^2+^) cations, and ammonia, effectively suppresses a wide range of disease-causing soilborne pathogens (16–19), and several soil physicochemical and microbial characteristics, such as the pH, electrical conductivity (EC), organic carbon content, microbial population, activity, and composition, are improved (20–22). Therefore, RSD has received considerable attention as an alternative to chemical fumigation in Japan, the United States, and China (16–19). Furthermore, recent studies have indicated that RSD-treated soils still possess the ability to suppress diseases, even under conditions of equal pathogen abundance with diseased soils achieved by pathogen reinoculation after disinfestation (23, 24). Thus, important changes in soils with biotic or abiotic properties, other than those relating to the decrease in pathogen abundance, ultimately responsible for disease suppression, should be uncovered in the RSD-treated soils.

**FIG 1:**
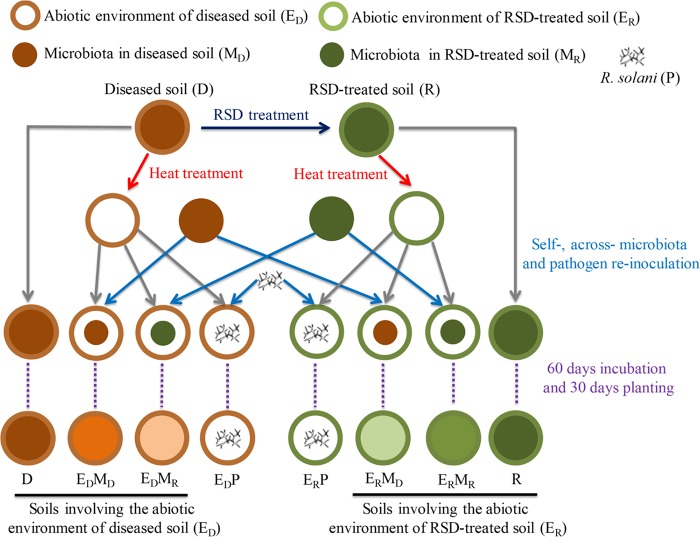
Schematic diagram of the experimental design. The diseased soil (D) infested with R. solani was RSD treated, which involved the addition of alfalfa and maintenance of soil anaerobic conditions for 18 days. The naturally drained RSD-treated soil was defined as R soil. Subsequently, the D and R soils were heat treated at 80°C, which decreased the abundances of bacterial and fungal more than 99.8%. Thereafter, the heat-treated diseased soil was reinoculated with self-microbiota (10% raw D soil, wt/wt, defined as E_D_M_D_), cross-microbiota (10% raw R soil, wt/wt, defined as E_D_M_R_), or pathogen (R. solani, defined as E_D_P). Similarly, the heat-treated R soil was also reinoculated with self- (E_R_M_R_), cross-microbiota (E_R_M_D_), or pathogen (E_R_P), respectively. After these soils were incubated for 60 days, pregerminated cucumber seeds were planted in these soils for 20 days. Soils involving the abiotic environment of the diseased soil (D, E_D_M_D_, and E_D_M_R_) and RSD-treated soil (R, E_R_M_D_, and E_R_M_R_) were aggregately named E_D_ and E_R_, respectively. Soils involving the initial microbiota of the diseased soil (D, E_D_M_D_, and E_R_M_D_) and RSD-treated soil (R, E_D_M_R_, and E_R_M_R_) were aggregately named M_D_ and M_R_, respectively. Detailed descriptions of the abbreviations for these soils are listed in Table 1.

Rhizoctonia solani Kühn, a widespread soilborne pathogen, infects a wide range of host plant species, such as agricultural and horticultural crops, and is responsible for economically important crop damage and yield losses. Cucumber (Cucumis sativus L.) R. solani damping-off diseased soil and RSD-treated diseased soil are considered two original soils in this study. Heat treatment and pathogen or soil microbiota self- and cross-reinoculations created diverse microbial communities in the two types of soil (Fig. 1 and Table 1), and the disease-suppressive abilities of these microbial communities were tested in a pot experiment. The aims were to answer the following: (i) how soil environmental factors and introduced microbiota determine the reassembly of microbial communities and disease suppression in these artificially managed soils, (ii) what the critical disease-suppressive agents are, and (iii) what the characteristics of these suppressive agents are. Moreover, the disease-suppressive function and the environment-dependent characteristics of a representative agent, *Zopfiella*, were further validated.

**TABLE 1:** The detailed description of the soils treated during experimental design

Abbreviation	Description
D	Diseased soil infested by the pathogen R. solani
E_D_M_D_	Heat-treated diseased soil reinoculated with raw diseased soil (10%, wt/wt)
E_D_M_R_	Heat-treated diseased soil reinoculated with soil (10%, wt/wt) that had been subjected to RSD^*a*^
E_D_P	Heat-treated diseased soil reinoculated with R. solani
R	Diseased soil treated by RSD
E_R_M_D_	Heat-treated R soil reinoculated with raw diseased soil (10%, wt/wt)
E_R_M_R_	Heat-treated R soil reinoculated with raw R soil (10%, wt/wt)
E_R_P	Heat-treated R soil reinoculated with R. solani
E_D_	Soils involving the abiotic environment of diseased soil (D, E_D_M_D_, and E_D_M_R_)
E_R_	Soils involving the abiotic environment of RSD-treated soil (R, E_R_M_D_, and E_R_M_R_)
M_D_	Soils involving the initial microbial community of diseased soil (D, E_D_M_D_, and E_R_M_D_)
M_R_	Soils involving the initial microbial community of RSD-treated soil (R, E_D_M_R_, and E_R_M_R_)

a RSD, reductive soil disinfestation.

## RESULTS

### Abundance of R. solani and damping-off disease indices.

**FIG 2:**
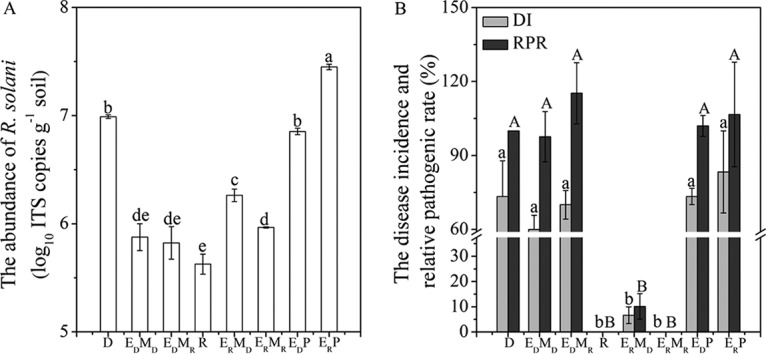
R. solani abundance (A) and disease incidence (DI) and relative pathogenic rate (RPR) (B) in the different soils after planting. DI = (number of infected plants in a replicate/10) × 100; RPR = [(DI in each replicate/abundance of R. solani in that replicate)/(DI in D soil/abundance of R. solani in D soil)]. Error bars indicate SEs, and different letters indicate significant differences according to Duncan’s test (*P* < 0.05).

A schematic diagram of the experimental design and detailed definitions of the abbreviations used for treated soils are shown in Fig. 1 and Table 1. After planting, the R. solani abundances in D, E_D_P, and E_R_P soils were 9.84 × 10^6^, 6.85 × 10^6^, and 7.45 × 10^7^ copies g^−1^, respectively, which were significantly (*P* < 0.05) higher than those in the other soils, in which the R. solani abundances ranged from 4.40 × 10^5^ to 1.88 × 10^6^ copies g^−1^ (Fig. 2A). The disease incidence (DI) in the E_D_ (D, E_D_M_D_, and E_D_M_R_) soils was significantly (*P* < 0.01) higher than those in the E_R_ (R, E_R_M_D_, and E_R_M_R_) soils, and cucumber seedlings in the heat-treated and pathogen-reinoculated soils (E_D_P and E_R_P) were largely infected (Fig. 2B). The relative pathogenic rate (RPR), which was defined as the pathogenic ability per unit of pathogen abundance, showed a trend similar to that of the DI in these soils (Fig. 2B). Furthermore, the DI and RPR in E_R_M_D_ soil were higher than those in R and E_R_M_R_ soils, although the results were not statistically significant (*P* > 0.05).

### Microbial abundance and alpha diversity.

The abundances of bacteria and fungi were significantly (*P* < 0.05) higher in the E_R_ (R, E_R_M_D_, and E_R_M_R_) soils (2.78 × 10^10^ to 4.64 × 10^10^ and 1.90 × 10^9^ to 3.08 × 10^9^ copies g^−1^, respectively) than those in the E_D_ (D, E_D_M_D_, and E_D_M_R_) soils (1.19 × 10^10^ to 1.48 × 10^10^ and 1.78 × 10^8^ to 6.80 × 10^8^ copies g^−1^, respectively) after planting (Fig. S1). The R soil considerably (*P* < 0.05) reduced fungal observed species numbers, as well as their diversity and evenness, compared with those in D soil (Table S1). Furthermore, Chao and Shannon indices for bacteria and fungi in the heat-treated soils were significantly lower (*P* < 0.05) than those for the non-heat-treated soils (E_D_M_D_ or EDMR versus D; E_R_M_D_ or ERMR versus R).

### Soil microbial community and environmental factors.

**FIG 3:**
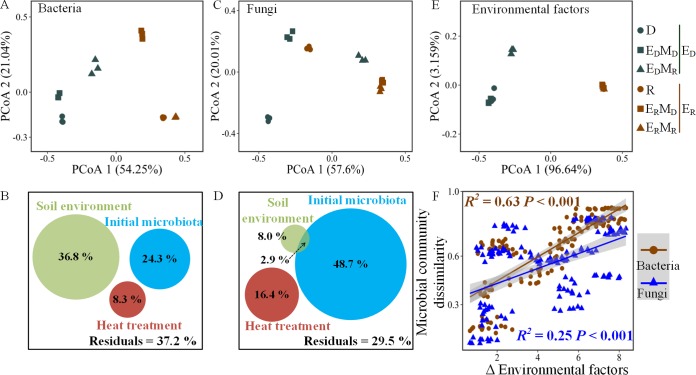
Dissimilarities in the soil microbial community and their contributors. (A and C) Principal-coordinate analyses (PCoA) based on soil bacterial (A) and fungal (C) OTU distributions using the Bray-Curtis indices. The green shapes represent E_D_ soils involving the abiotic environment of the diseased soil, and the brown shapes represent E_R_ soils involving the abiotic environment of RSD-treated soil. (B and D) Contributions of the soil environment (E_D_ containing D, E_D_M_D_, and EDMR versus ER containing R, E_R_M_D_, and E_R_M_R_), initial microbiota (M_D_ containing D, E_D_M_D_, and ERMD versus MR containing R, E_D_M_R_, and E_R_M_R_), and heat treatment (heat-treated soils containing E_D_M_D_, E_D_M_R_, E_R_M_D_, and ERMR versus non-heat-treated soils containing D and R) on the assembly of bacterial (B) and fungal (D) communities calculated based on variance partitioning analyses. (E) PCoA based on the soil environmental factors (consisting of soil physicochemical properties listed in Table S3 and S4). (F) Correlations between the microbial community dissimilarities and the differences in the environmental factors. Δ Environmental factors were calculated based on the z-score normalized to the physicochemical properties using Euclidean indices.

RSD treatment significantly (*P* < 0.01, permutational multivariate analysis of variance [PERMANOVA]) altered the bacterial and fungal communities, and heat treatment followed by microbiota reinoculation, especially microbiota cross-reinoculation, also changed the bacterial and fungal communites (*P* < 0.01) (Fig. 3A and C). The dissimilarity in the bacterial community was primary caused by differences in the soil environment, followed by the initial microbiota, whereas the dissimilarity in the fungal community was mainly caused by difference in the initial microbiota (Fig. 3B and D). In addition, RSD treatment significantly (*P* < 0.05) affected several physicochemical properties by increasing the soil pH and the contents of several carbon fractions and by decreasing the soil electrical conductivity (EC) and inorganic nitrogen content (Tables S2 and S3), whereas heat treatment and microbiota reinoculation rarely affected the soil physicochemical properties compared with those in the nonheat-treated soils (Fig. 3E). Furthermore, the pairwise Bray-Curtis indices (Fig. 3F and Fig. S2A to D) and beta nearest taxon indices (βNTI) (Fig. S2E to H) for bacterial and fungal communities positively correlated with the pairwise distances of the soil environmental factors consisting of several physicochemical properties.

### Soil microbial community composition.

**FIG 4:**
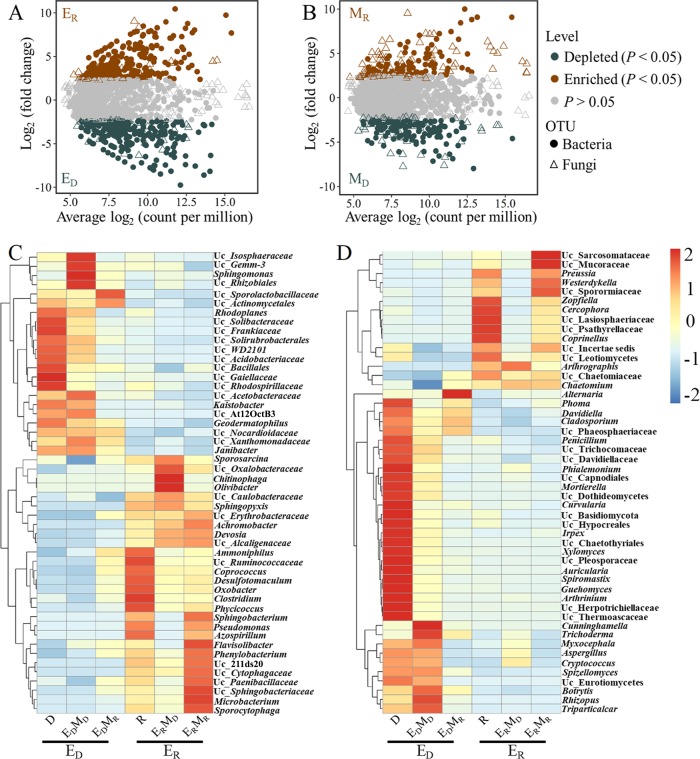
Microbial composition of the different soils at multiple levels. (A and B) Abundance patterns of bacterial and fungal OTUs in E_D_ (D, E_D_M_D_, and E_D_M_R_) and E_R_ (R, E_R_M_D_, and E_R_M_R_) soils (A) and M_D_ (D, E_D_M_D_, and E_R_M_D_) and M_R_ (R, E_D_M_R_, and E_R_M_R_) soils (B). The OTUs that were significantly (likelihood ratio test, *P* < 0.05, corrected for false-discovery rate [FDR]) different in abundance between E_D_ and E_R_ or M_D_ and M_R_ are colored. The *x* axis reports the average OTU abundance (as counts per million [CPM]), and the *y* axis reports the log_2_ fold change (E_R_ to E_D_ and M_R_ to M_D_). (C and D) Heat maps displaying the top 50 bacterial (C) and fungal (D) genera that were significantly (*P* < 0.05) different in relative abundance between E_R_ and E_D_ soils. The color scale indicates the relative values of the abundance of each genus across the different soils. The taxonomic name following “Uc_” represents the most detailed classification of the currently unclassified genera.

E_D_ (D, E_D_M_D_, and E_D_M_R_) and E_R_ (R, E_R_M_D_, and E_R_M_R_) (Fig. 4A) or M_D_ (D, E_D_M_D_, and E_R_M_D_) and M_R_ (R, E_D_M_R_, and E_R_M_R_) (Fig. 4B) soils respectively harbored distinct bacterial and fungal community compositions with specific sets of operational taxonomic units (OTUs). Linear discriminant analysis (LDA) effect size (LEfSe) analysis revealed that the microbial composition significantly varied between E_D_ and E_R_ soils at multilevel taxa, such as the bacterial orders *Sphingobacteriales*, *Clostridiales*, and *Burkholderiales* and the fungal order Sordariales (Fig. S3). Furthermore, the top 50 bacterial and fungal genera that significantly differed between E_D_ and E_R_ soils were also identified, and we also found several genera whose relative abundances were significantly different between E_R_M_D_ and E_R_M_R_ soils, such as *Sphingobacterium*, *Pseudomonas*, *Zopfiella*, and Uc_Sarcosomataceae (currently unclassified genera belonging to Sarcosomataceae) (Fig. 4C and D). In addition, similarity percentage (SIMPER) analysis revealed these biomarkers (at the genus level) that largely contributed to the differences between E_D_ and E_R_ soils where Uc_*Xanthomonadaceae*, *Sphingobacterium*, *Chitinophaga*, Uc_*Nocardioidaceae*, Uc_*Sphingobacteriaceae*, Uc_Chaetomiaceae, *Zopfiella*, and Uc_Lasiosphaeriaceae were the dominat (relative abundance larger than 6% in at least one soil) bacterial and fungal genera (Table 2).

**TABLE 2:** SIMPER analysis showing the top contributors (contribution > 1%) to the dissimilarity in the microbial community between E_D_ and E_R_ soils

Organsim type	Biomarker genus^*a*^	Avg abundance (%)^*b*^						Contribution to dissimilarity (%)	Cummulative contribution to dissimilarity (%)	*P* value^*c*^
		E_D_			E_R_					
		D	E_D_M_D_	E_D_M_R_	R	E_R_M_D_	E_R_M_R_			
Bacteria	**Uc_*Xanthomonadaceae***	11.67	16.87	10.93	0.56	3.39	0.39	6.86	10.52	0.001
	***Sphingobacterium***	0.01	0.00	0.09	9.54	0.01	12.51	4.26	23.84	0.005
	***Chitinophaga***	0.02	0.01	0.01	0.83	19.09	0.58	4.07	30.08	0.001
	**Uc_*Nocardioidaceae***	7.38	7.18	6.69	2.25	1.56	3.04	2.88	34.48	0.001
	**Uc_*Sphingobacteriaceae***	0.01	0.01	2.32	3.97	2.00	9.06	2.58	38.43	0.001
	Uc_*Sporolactobacillaceae*	2.49	2.08	4.80	0.65	0.16	0.17	1.44	40.63	0.001
	Uc_*Ruminococcaceae*	0.16	0.06	2.10	5.22	1.67	1.81	1.37	44.86	0.016
	Uc_*Gaiellaceae*	5.48	0.94	0.24	0.33	0.05	0.05	1.32	46.88	0.007
	Uc_*Solirubrobacterales*	3.32	2.42	0.85	0.38	0.08	0.04	1.31	48.89	0.001
	*Microbacterium*	0.06	0.04	0.35	1.57	0.91	3.78	1.30	50.88	0.001
	*Kaistobacter*	3.30	2.76	0.28	0.27	0.47	0.03	1.16	54.51	0.011
	Uc_*Erythrobacteraceae*	0.06	0.03	1.30	2.05	1.94	2.46	1.05	57.85	0.001
Fungi	**Uc_Chaetomiaceae**	11.14	8.52	23.09	35.90	25.72	30.05	8.22	14.87	0.003
	***Zopfiella***	0.35	0.01	0.35	6.43	0.24	3.60	1.63	76.28	0.011
	**Uc_Lasiosphaeriaceae**	0.46	0.01	0.27	6.56	0.28	2.56	1.48	79.18	0.009

a The bacterial and fungal genera that significantly varied between E_D_ and E_R_ soils are listed. The taxonomic name of the genus with a relative abundance greater than 6% in at least one soil is in bold.

b Average abundance from 3 replicates.

c *P* values were calculated by 999 permutations. The treatment abbreviations are defined in Fig. 1 and Table 1.

In addition, according to their distributions in E_D_ and E_R_ soils, microbes could be classified into three typical groups, namely, microbes having a broad environmental adaptation range covering two types of soil environments that could be mostly transferred from each other, such as those having no significant differences in abundance between E_D_ and E_R_ soils (group I); microbes preferring or only favoring the abiotic environment of RSD-treated soils that could not be or partly be transferred from RSD-treated soils to the diseased soils, such as *Zopfiella*, Uc_Lasiosphaeriaceae, *Sphingobacterium*, and Uc_*Sphingobacteriaceae*, whose abundances were significantly higher in E_R_ soils (group II); and, conversely, microbes restricted to conditions in the diseased soils that could not be or partly be transferred from the diseased soils to the RSD-treated soils, such as Uc_*Xanthomonadaceae* and Uc_*Nocardiodaceae* (group III).

### Associations among biomarkers, disease incidence, and soil environmental factors.

**FIG 5:**
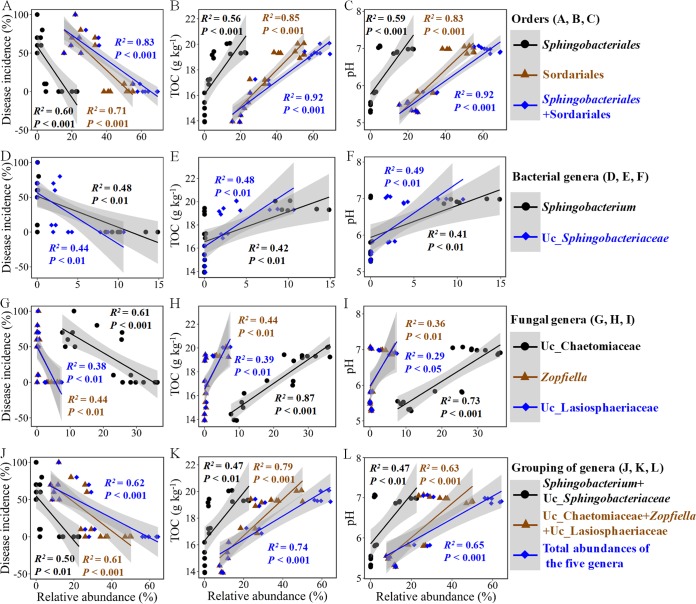
Potential disease-suppressive agents and their associated environmental factors. The dominant bacterial and fungal taxa, filtered by relative abundances greater than 6% in at least one soil and contributing more than 1% in SIMPER analysis, significantly (*P* < 0.05) negatively correlated with the disease incidence are involved here, i.e., *Sphingobacterium* and Uc_*Sphingobacteriaceae*, belonging to the bacterial order *Sphingobacteriales*, and Uc_Chaetomiaceae, *Zopfiella*, and Uc_Lasiosphaeriaceae, belonging to the fungal order Sordariales. TOC, soil total organic carbon.

Regression analyses revealed that the relative abundances of the bacterial order *Sphingobacteriales* and the fungal order Sordariales were significantly (*P* < 0.001) and negatively correlated with the DI (Fig. 5A). Besides, the relative abundances of the bacterial genera *Sphingobacterium* and Uc_*Sphingobacteriaceae*, within the order *Sphingobacteriales*, and the fungal genera Uc_Chaetomiaceae, *Zopfiella*, and Uc_Lasiosphaeriaceae, within the order Sordariales, as well as the grouping of these genera were significantly (*P* < 0.01) negatively correlated with the DI (Fig. 5D, G, and J). Furthermore, the relative abudances of these potential disease-suppressive agents significantly (*P* < 0.05) positively correlated with the soil pH and total organic carbon (TOC) content (Fig. 5B, C, E, F, H, and I).

### Validation of the representative disease-suppressive agent.

**FIG 6:**
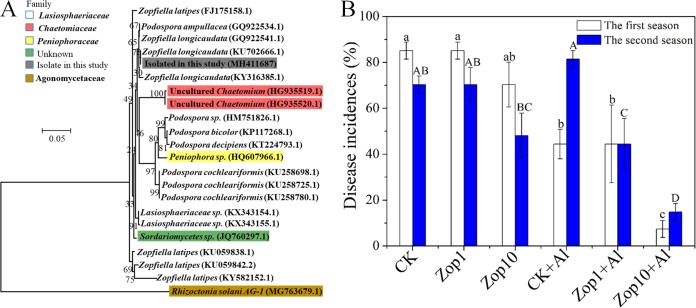
Validation of the representative disease-suppressive agent *Zopfiella*. (A) Phylogenetic tree based on the isolated *Zopfiella* sp. Shown are the ITS sequence and the best matches in NCBI using the neighbor-joining method. (B) Cucumber damping-off disease incidences in the verification test. CK and CK+Al represent the diseased soil and alfalfa-amended diseased soil, respectively; Zop1 and Zop10 represent the diseased soil inoculated with 0.75 and 7.5 g of *Zopfiella* sp. mycelia per kg of soil, respectively; Zop1+Al and Zop10+Al represent the alfalfa-amended diseased soil inoculated with 0.75 and 7.5 g of *Zopfiella* sp. mycelia per kg of soil. Error bars indicate SEs, and different letters indicate significant differences according to Duncan’s test (*P* < 0.05).

To validate the potential disease-suppressive agent, a representative fungal strain was isolated from R soil and identified as a *Zopfiella* sp. according to its morphology and internal transcribed spacer (ITS) sequence (Fig. 6A). We found that the sole inoculation of 0.75 (Zop1) and 7.5 (Zop10) g of *Zopfiella* sp. per kg of soil in the diseased soil (CK) did not significantly (*P* > 0.05) prevent the development of damping-off disease during the successive cropping seasons, although the DI in the Zop10 soil was lower than that in the CK (Fig. 6B). In contrast, the inoculation of 0.75 g of *Zopfiella* sp. per kg of soil combined with the alfalfa amendment (Zop1+Al) had no effect on DI during the first growth season compared with alfalfa-amended CK soil (CK+Al); however, it resulted in a significant (*P* < 0.05) decrease in the DI during the second season. Furthermore, soil inoculation with 7.5 g of *Zopfiella* sp. per kg of soil combined with alfalfa amendment (Zop10+Al) considerably (*P* < 0.05) prevented cucumber damping-off disease during both growth seasons (Fig. 6B).

## DISCUSSION

Soilborne diseases cause significant economic damage to crops, and some soil management strategies have been developed, such as organic amendment and RSD (15, 25), to control their development. However, the underlying mechanisms have not been clarified. In addition, studies have aimed to develop physicochemical and microbial indicators of disease suppression in soil, but the reliability of these indicators has been shown to be inconsistent (26–28). Several studies shown that the physicochemical properties of soil may contribute to disease suppression (27), including the soil pH and salinity (indicated by EC) (28). However, we found that heat-treated and pathogen-reinoculated RSD soil (E_R_P), with physicochemical properties similar to but microbiota different from those of R soil, did not prevent disease, indicating that the biotic properties of the RSD-treated soil, rather than the abiotic properties, may be directly responsible for disease suppression. Furthermore, it has long been believed that the inoculum level of soilborne pathogens is the key determinant of disease occurrence (29). Consistent with previous reports (19–21), we found that RSD significantly decreased the R. solani abundance in soil, which largely explains the prevention of damping-off disease by RSD in previous studies. However, the abundances of R. solani in this study were approximately equal in all of the soils, except for D, E_D_P, and E_R_P, whereas the DIs were dramatically different, indicating that the relative pathogenic abilities of these soils per unit of R. solani were different. Thus, other critical disease-suppressive agents determining the DI, in addition to the decrease in the abundance of R. solani, should be present in the microbiota of the RSD-treated soils.

It is well accepted that the abiotic environment of the soil greatly determines its inhabitants (30). In this study, we found that the abiotic environmental factors, including multiple physicochemical properties, such as the pH and the contents of carbon fractions, highly influenced the dissimilarity (based on the Bray-Curtis indices) and the assembly (based on the βNTI) of the microbial community, especially the bacterial community, which is in line with previous reports (30–32). In contrast, the influence of the abiotic environment on the assembly of the fungal community has been explored less in previous studies, possibly because the fungal ITS sequence, which is mostly used in studies of the fungal community, has a lower resolution than the bacterial 16S rRNA gene and could not be aligned readily. In spite of this, we found in this study that the fungal community was less dependent on the soil abiotic environment than the bacterial community (based on both the Bray-Curtis indices and the βNTI), whereas the initial microbiota before the environmental change highly affected the established fungal community.

In this study, disease suppression was not transferable from RSD-treated soil to diseased soil through microbial community exchange processes. Therefore, we concluded that disease suppression agents existed in group II. In contrast, Mendes et al. (11) and Cha et al. (12) reported that disease suppression agents (*Gamaproteobacteria* and *Streptomyces*, respectively) could be transferred from disease-suppressive soils to disease-conducive soils through microbial community exchange processes similar to those reported here. We inferred that the transfer of microbial species from one soil to another depended on its range of environmental adaptation and the degree of environmental difference between the two soils. Differences in the soil pH, which is considered the primary environmental determinant of bacterial distribution (31, 32), were small between disease-suppressive and diseases-conducive soils in studies by Mendes et al. (11) and Cha et al. (12) (0.2 and 0.17, respectively). It is highly possible that the suppressive agents adapted to both the abiotic environments of disease-suppressive and -conducive soils, and could, theoretically, transfer from one to the other in these studies. Reflecting on this study, the differences in the soil environmental factors between the diseased and RSD-treated soils, such as the pH and carbon quality and quantity, may have been large enough to exceed the adaptation ranges of the suppressive agents that may have ultimately induced the nontransferability of the disease suppression capability. This interpretation is further supported by the fact that disease suppression markedly declined when the abiotic environment, such as the pH, of the disease-suppressive soil changed greatly (33, 34). Additionally, we also found that many microbial species, such as *Zopfiella* and *Sphingobacterium*, were highly influenced by reinoculated microbiota, where their relative abundances in the reestablished soil was associated with their initial abundances in the inoculants. We contend that the disease-suppressive microbial agents may have been at least partly influenced by the reinoculated microbiota, considering that the DI in E_R_M_D_ soil was slightly higher than in R and E_R_M_R_ soils.

Further analyses showed that the orders *Sphingobacteriales* and Sordariales, and their affiliated genera *Sphingobacterium*, Uc_*Sphingobacteriaceae*, Uc_Chaetomiaceae, *Zopfiella*, and Uc_Lasiosphaeriaceae, negatively correlated with the DI, and consequently were potential disease suppression agents. Microorganisms within *Sphingobacteriaceae* do not tolerate acidic environments and can produce antifungal compounds, such as indophenol oxidase, hydrogen sulfide, and proteolytically active enzymes, by decomposing carbohydrates (35, 36). Furthermore, it has been reported that many microbial species in the family of Chaetomiaceae produce carbohydrate-active enzymes (37) and prevent soilborne pathogens and diseases (38, 39). As a genus of fungi belonging to the same order (Sordariales) as Chaetomiaceae, *Zopﬁella* produces the antifungal compound zopfiellin, which acts against plant pathogens (40, 41). It is sometimes replaced by the closely related but currently undistinguished *Podospora* (42), which isprevalent in RSD-treated soils (19, 21, 24, 43). Thus, we hypothesized that increases in these disease suppression agents drive disease suppression, and specific environment associations, such as Uc_Sphingobacteriaceae with the neutral soil pH and Uc_Chaetomiaceae, *Zopfiella*, and Uc_Lasiosphaeriaceae with the available carbon sources, determined that the transfer from the RSD-treated soil to the diseased soil would not occur. In attempting to validate the hypothesis, we found that inoculation with *Zopfiella* accompanied by organic carbon amendment prevented damping-off disease and the level of inoculum affected the control efficiency. These results support previous inferences that disease suppression agents are environment dependent and influenced by reinoculated microbiota. Furthermore, the nontransferability of the disease suppression ability from the RSD-treated soil to the diseased soil could be explained by the fact that organic amendment in the RSD treatment increased the total microbial abundance or activity in the soil and thus prevented disease. This seemingly coincides with general disease suppression with the concept that the total amount of microbial abundance or activity contributes to disease suppression, but this is not well-understood currently (44). However, previous studies have also demonstrated that microbial abundance and activity are not always related to disease suppression (26, 45), indicating that the specific microbial composition is important even in general disease suppression. Furthermore, we found that the organic amendment without *Zopfiella* could not prevent disease, which indicates that both the specific microbial agent and its adapted environment are essential for disease suppression in RSD-treated soil.

Overall, studies on disease-suppressive soils, especially specific suppressive soils, have provided a framework in which most defined systems have identified specific microbial species as the primary factors in disease suppression. However, the matched abiotic soil environment with these microbial agents is usually neglected (44). In this study, we deciphered the mechanism of disease suppression in an artificially managed disease-suppressed soil through systematic comparision of the disease suppression abilities of various microbial communities harbored by two types of abiotic environments. We validated that both biotic agents and their adapted abiotic environment were important for disease suppression. Furthermore, this study provided a systematic procedure for characterizing disease-suppressive agents in an artificially managed soil, and these results will gradually close the knowledge gap regarding soil environment-microbe interactions.

## MATERIALS AND METHODS

### Description of diseased soil and reductive soil disinfestation.

Salinized and acidified soil containing the damping-off disease-causing pathogen R. solani was collected from a greenhouse located in Changzhou (32°04′N, 120°12′E), Jiangsu Province, China. Crop residues and stones were removed from the soil by passing through a 2-mm sieve. The characteristics of the soil were as follows: moisture content of 14.9%, pH of 5.26, EC of 0.89 mS cm^−1^, TOC content of 18.11 g kg^−1^, total nitrogen (TN) content of 2.44 g kg^−1^, nitrate (NO_3_^−^-N) content of 592 mg kg^−1^, and 1.36 × 10^7^ ITS copies of R. solani g^−1^. Alfalfa (Medicago sativa), composed of a TOC content of 399.1 g kg^−1^ and a TN content of 13.45 g kg^−1^, was powdered and used as the organic matter of the RSD treatment. RSD treatment was performed in a box (length by width by height = 25 by 25 by 25 cm), where the diseased soil was combined with 2% (wt/wt) alfalfa, irrigated to saturation, and sealed with a plastic film. The untreated diseased soil and the RSD-treated soil were considered two original soils, defined as D and R soils, respectively. The D and R soils were incubated for 18 days at 35°C and then respectively drained, passed through a 2-mm sieve, and homogenized for the next step of the experiment.

### Experimental design and cultivation of cucumber seedlings.

The schematic diagram of the experimental design is listed in Fig. 1. There were three replicates of eight treatments using a completely randomized design, where each replicate in one treatment contained 10 culture bottles (neck diameter by bottom diameter by height = 7 by 8 by 12 cm; volume = 500 ml). Briefly, treatments were composed of non-heat-treated diseased soil (D) and RSD-treated (R) soil and heat-treated (80°C for 2 h to reduce microbial abundance by >99.8%) D and R soils reinoculated with 10% (wt/wt) of raw D soil (E_D_M_D_ and E_R_M_D_, respectively), 10% (wt/wt) of raw R soil (E_D_M_R_ and E_R_M_R_, respectively), or a suspension of R. solani mycelium (E_D_P and E_R_P, respectively) to the levels present in D soil detected based on real-time quantitative PCR (qPCR). Soils involving the abiotic environment of the diseased soil (D, E_D_M_D_, and E_D_M_R_) and RSD-treated soil (R, E_R_M_D_, and E_R_M_R_) were aggregately defined as E_D_ and E_R_, respectively. Soils contained the initial microbiota of the diseased soil (D, E_D_M_D_, and E_R_M_D_) and RSD-treated soil (R, E_D_M_R_, and E_R_M_R_) were aggregately defined as M_D_ and M_R_, respectively.

All of the culture bottles were incubated for 60 days at 20°C and irrigated with 5 ml of sterile water every 10 days (approximately 15% soil water content). After incubation, approximately 10 g of soil was collected from each bottle, and those soils from 10 bottles in a replicate were mixed to form a composite biological replicate (this time point was defined as “after incubation”), and a single, pregerminated cucumber seed (cv. JinChun 5) was placed in each bottle and cultivated for 20 days, with average day and night temperatures of 30 and 18°C, respectively, before the soil was mixed thoroughly and collected for analysis (this time point was defined as “after planting”). The disease indices, including DI and RPR, were respectively calculated using the following formulae: DI = (the number of infected plants in a replicate/10) × 100; RPR = [(DI in each replicate/the abundance of R. solani in that replicate)/(DI in D soil/the abundance of R. solani in D soil)] × 100.

### Measurement of physicochemical soil properties.

We quantified the soil physicochemical properties as surrogate measures of the abiotic environmental factors. The soil pH and EC were measured in slurries (soil/water, 1:2.5 and 1:5, wt/vol, respectively) using a S220K pH meter (Mettler-Toledo International Inc., Shanghai, China) and a conductivity meter (DDS-320; Dapu Instrument Co., Ltd., Shanghai, China), respectively. The soil TOC content was measured using wet digestion with H_2_SO_4_-K_2_Cr_2_O_7_ (46), and fractions of easily oxidized organic carbon (EOC) content were oxidized using 333 mmol liter^−1^ (EOC_333_), 167 mmol liter^−1^ (EOC_167_), and 33.3 mmol liter^−1^ (EOC_33.3_) of KMnO_4_, following the method reported by Blair et al. (47). The soil inert organic carbon content (IOC) was equal to the TOC content minus EOC_333_ content. The light fraction organic carbon (LFOC) content was measured using the approach described by Compton and Boone (48), and the heavy fraction organic carbon (HFOC) content was equal to the TOC content minus LFOC content. Total organic nitrogen (TON) was determined using semi-micro-Kjeldahl digestion (49). NO_3_^−^-N and ammonium (NH_4_^+^-N) were extracted using 2 mol liter^−1^ of KCl solution (1:5 wt/vol), followed by shaking at 250 revolutions min^−1^ for 1 h and filtering for 30 min. The content was measured using a continuous flow analyzer (San++; Skalar Analytical B.V., Breda, The Netherlands).

### Microbial quantification.

Microbial DNA was extracted from 0.25 g of soil using the PowerSoil DNA isolation kit (MO BIO Laboratories, Inc., USA), according to the manufacturer’s instructions. Real-time PCR amplification was carried out in 8-well tubes using the CFX96 real-time system (Bio-Rad Laboratories Inc., Hercules, CA); qPCR mixtures and thermal conditions followed those reported by Huang et al. (20). The bacterial (Eub338/Eub518), fungal (ITS1-f/5.8s), and R. solani (ST-RS1/ITS4R) primer sets are listed in Table 3. Melting curves were recorded to evaluate the amplification specificity at the end of each PCR run.

**TABLE 3:** Amplification primers used in this study

Target genes	Primer	Sequence (5′–3′)	Reference
Bacterial 16S rRNA	Eub338	CCTACGGGAGGCAGCAG	61
	Eub518	ATTACCGCGGCTGCTGG	62
	515F	GTGCCAGCMGCCGCGG	63
	907R	CCGTCAATTCMTTTRAGTTT	64
			
Fungal ITS	ITS1f	TCCGTAGGTGAACCTGCGG	65
	5.8s	CGCTGCGTTCTTCATCG	66
	ITS3	GCATCGATGAAGAACGCAGG	67
	ITS4R	TCCTCCGCTTATTGATATGC	65
			
R. solani ITS	ST-RS1	AGTGTTATGCTTGGTTCCACT	68
	ITS4R	TCCTCCGCTTATTGATATGC	65

With the exception of E_D_P and E_R_P soils, microbial DNA from soil samples (*n* = 18) collected after planting was sequenced using individual barcoded primers 515F and 907R and primers ITS3F and ITS4R (Table 3) to amplify the V4-V5 region (365 to 375 bp) of the bacterial 16S rRNA genes and the fungal ITS region (191 bp), respectively, using reaction mixtures and thermal conditions described by Zhao et al. (50). After amplification, the PCR products were purified with AgencourtAMPure XP beads (Beckman Coulter, CA) and adjusted to equimolar concentrations. The paired-end sequencing was carried out using the Illumina MiSeq system (USA) at Genesky Biotechnologies, Inc. (Shanghai, China).

### Bioinformatic analyses.

The scripts for processing the raw sequencing data are listed in the supplemental material. Briefly, sequencing data were processed using the QIIME software package (version 1.9.1), following the approach described by Caporaso et al. (51), where paired-end FASTQ sequences of the raw sequence data were merged using the default arguments in multiple_join_paired_ends.py. Subsequently, multiple_extract_barcodes.py and multiple_split_libraries_fastq.py were used to remove the barcode sequences and control the sequence quality, respectively. Thereafter, pick_open_reference_otus.py was used to cluster the quality-filtered sequences to operational taxonomic units (OTUs) at 97% similarity and annotate them according to the Greengenes 13_8 database (bacteria) (52) and UNITE databse (fungi) (53). Parallel_inentify_chimeric_seqs.py and Usearch –uchime2 were used to identify bacterial and fungal chimeric OTUs, respectively, and then filter_otus_from_otu_table.py was used to filter these chimeric OTUs from the OTU tables. Finally, the bacterial and fungal sequences were rarefied to 26,000 and 45,000 for all soil samples, respectively. The default arguments in alpha_diversity.py, based on the rarefied OTU tables, were performed to analyze the microbial alpha-diversity. The default arguments in make_phylogeny.py in QIIME and the neighbor-joining method in MEGA, based on the representative sequences of the bacterial and fungal OTUs, were used to generate the phylogenetic trees, respectively.

### Validation of the representative disease-suppressive agent.

Based on the analysis of the aforementioned results, we tested the function of the *Zopfiella* sp. as a potential disease-suppressing agent. First, the strain was isolated from R soil using gradient dilution coating in soil extract agar medium (54) and identified according to its ITS sequence and morphology. The cultivation of *Zopfiella* sp. mycelia was performed in liquid potato dextrose medium at 28°C for 7 days. The mycelia were then collected, weighed, and homogenized in sterilized water. Three replicates of six treatments, which were composed of diseased soil (CK), diseased soil inoculated with 0.75 or 7.5 g of *Zopfiella* sp. mycelia per kg of soil (Zop1 or Zop10, respectively), diseased soil amended with 2% (wt/wt) alfalfa (CK+Al), and CK+Al inoculated with 0.75 or 7.5 g of *Zopfiella* sp. mycelia per kg of soil (Zop1+Al or Zop10+Al, respectively), were arranged in a completely randomized design. Treatment boxes (25 by 25 by 6 cm) containing 2.5 kg of soil were planted with 9 pregerminated cucumber seeds and cultivated as previously described. After 20 days, the DI was recorded, the seedlings were removed, and the soil in each box was mixed in preparation for a repeat cultivation of the cucumber seedlings.

### Data analysis.

Microbial count data were log_10_ transformed prior to statistical analysis. The treatment effects were tested using one-way analysis of variance (ANOVA) and Duncan’s test (*P* < 0.05) using SPSS 19.0 software (SPSS Inc., Chicago, IL). The treatment effects (excluding E_D_P and E_R_P) on the soil environmental factors and microbial community were estimated by principal-coordinate analysis (PCoA) using the *pco* function within the R package *labdsv* (55). Permutational multivariate analysis of variance (PERMANOVA) based on Bray-Curtis index matrices to test for environmental factor and microbial community treatment differences was performed using the *adonis* function within the R package *vegan* (56). The contributions of the soil environment, initial microbiota, and heat treatment to microbial community dissimilarities were tested using variance partitioning analysis in the *varpart* function within the *vegan* package (56). Dissimilarities in the microbial community based on the Bray-Curtis indices were linearly regressed against differences in the soil environmental factors based on the Euclidean indices. The microbial beta nearest taxon indices (βNTI) were calculated to assess phylogenetic community assembly processes using the R package *picante* (57, 58). Briefly, pairwise βNTI values were calculated using the following formula: (observed β mean nearest taxon distance [βMNTD] − mean of the null distribution of MNTD)/standard deviation of the null distribution of MNTD. βNTI values were linearly regressed against differences in the soil environmental factors.

The microbial compositions between E_D_ (D, E_D_M_D_, and E_D_M_R_) and E_R_ (R, E_R_M_D_, and E_R_M_R_) soils and between M_D_ (D, E_D_M_D_, and E_R_M_D_) and M_R_ (R, E_D_M_R_, and E_R_M_R_) soils were compared using likelihood ratio tests (LRT) within the *edgeR* package (59), where the communities were expressed as relative abundance counts per million (CPM), normalized using the “trimmed means of M” (TMM) method, and filtered by abnegating the OTUs with a low relative abundance (sum of CPM < 9). Linear discriminant analysis Effect Size (LEfSe) analysis (http://huttenhower.sph.harvard.edu/galaxy) was further used to identify the microbial taxonomic differences (from phylum to family) between E_D_ and E_R_ soils. In addition, heat maps of top 50 bacterial and fungal genera significantly different between E_D_ and E_R_ soils were generated to visualize dissimilarities in the microbial composition using the R package *Pheatmap* (60). Similarity percentage (SIMPER) analysis to elucidate the biomarker genera to the differences between E_D_ and E_R_ soils was performed using the *simper* function within the R package *vegan* (56). Finally, linear regression analyses among the dominant and biomarker genera, disease incidence, and environmental factors were performed to identify the potential disease suppression agent and the adapted environmental factors.

### Accession number(s).

The raw sequencing data were deposited into the NCBI Sequence Read Archive (SRA) database (accession number SRP118835).

## Supplementary Material

Supplemental file 1
