# Supplementary material for: Characterizing the Key Agents in a Disease-Suppressed Soil Managed by Reductive Soil Disinfestation
Source: Appl Environ Microbiol. 2019 Mar 22;85(7):e02992-18. doi: 10.1128/AEM.02992-18 (PMC6585495; doi:10.1128/AEM.02992-18)
Supplement: Supplemental file 1 [file AEM.02992-18-s0001.pdf]

Supplementary material data to the article

Title: Characterizing the key agents in a disease-suppressed soil managed by reductive soil disinfestation

Running Title: Disease suppressive agent in artificially managed soil

Liangliang Liu[a†], Xinqi Huang[a†*], Jun Zhao[b,c], Jinbo Zhang[d], Zucong Cai[a,e]

[a] School of Geography Science, Nanjing Normal University, Nanjing 210023, China

[b] Jiangsu Center for Collaborative Innovation in Geographical Information Resource Development and Application, Nanjing 210023, China

[c] Key Laboratory of Virtual Geographical Environment (VGE), Ministry of Education, Nanjing Normal University, Nanjing 210023, China

[d] State Key Laboratory Cultivation Base of Geographical Environment Evolution, Nanjing 210023, China

[e] Jiangsu Provincial Key Laboratory of Materials Cycling and Pollution Control, Nanjing Normal University, Nanjing 210023, China

**Table S1.** The bacterial and fungal alpha diversities in the soils after cucumber seedlings planting

| Treatments | Bacteria | | | | Fungi | | | |
|---|---|---|---|---|---|---|---|---|
| | Chao | Observed species numbers | Shannon | Equitability | Chao | Observed species numbers | Shannon | Equitability |
| D | 3825±67 a | 2369±16 a | 8.57±0.01 a | 0.76±0.00 a | 1038±112 a | 834±57 a | 6.33±0.12 a | 0.65±0.01 a |
| $E_DM_D$ | 2890±64 c | 1625±27 c | 7.84±0.04 b | 0.73±0.00 b | 741±200 b | 503±117 c | 4.73±0.14 c | 0.52±0.01 bc |
| $E_DM_R$ | 3205±96 b | 1803±67 b | 7.70±0.15 b | 0.71±0.01 b | 553±7 c | 402±13 d | 4.67±0.09 c | 0.53±0.01 b |
| R | 4023±106 a | 2465±43 a | 8.74±0.08 a | 0.78±0.01 a | 930±39 a | 605±9 b | 5.06±0.07 b | 0.54±0.01 b |
| $E_RM_D$ | 2894±336 c | 1686±156 bc | 7.22±0.39 c | 0.67±0.02 c | 703±31 bc | 439±8 cd | 4.56±0.05 c | 0.52±0.00 c |
| $E_RM_R$ | 2979±162 bc | 1955±346 b | 7.76±0.15 b | 0.71±0.01 b | 628±46 bc | 397±3 d | 4.65±0.04 c | 0.54±0.00 b |

Values (means ± SD, $n = 3$) within the same column followed by different letters are significantly different at $P < 0.05$ according to Duncan's test.

The treatment abbreviations are defined in Figure 1 and Table 1.

**Table S2**. The contents of carbon fractions in the different soils after incubation and planting

| | Treatment | TOC ($g\ kg^{-1}$)[a] | $EOC_{333}$ ($g\ kg^{-1}$) | $EOC_{167}$ ($g\ kg^{-1}$) | $EOC_{33.3}$ ($g\ kg^{-1}$) | IOC ($g\ kg^{-1}$) | LFOC ($g\ kg^{-1}$) | HFOC ($g\ kg^{-1}$) |
|---|---|---|---|---|---|---|---|---|
| After incubation | D | 18.11±0.59 c | 3.34±0.21 d | 2.18±0.09 d | 1.32±0.03 b | 14.76±0.59 b | 4.22±0.57 c | 13.88±0.24 a |
| | $E_DM_D$ | 17.34±0.17 d | 3.57±0.04 d | 1.75±0.11 e | 1.24±0.07 b | 13.76±0.18 c | 5.07±0.97 c | 12.27±0.82 c |
| | $E_DM_R$ | 17.42±0.11 d | 3.49±0.17 d | 2.13±0.06 d | 1.30±0.03 b | 13.93±0.06 c | 4.76±0.49 c | 12.66±0.60 bc |
| | R | 21.71±0.10 a | 5.71±0.17 a | 3.11±0.19 a | 1.89±0.15 a | 16.00±0.26 a | 8.67±0.06 a | 13.04±0.08 abc |
| | $E_RM_D$ | 20.30±0.46 b | 4.22±0.27 c | 2.40±0.08 c | 1.86±0.11 a | 16.07±0.30 a | 6.91±1.24 b | 13.38±0.78 ab |
| | $E_RM_R$ | 20.74±0.44 b | 4.72±0.23 b | 2.84±0.07 b | 1.79±0.08 a | 16.02±0.22 a | 8.18±0.41 ab | 12.56±0.26 bc |
| After planting | D | 15.54±0.60 C | 2.75±0.20 B | 1.61±0.10 C | 1.05±0.04 D | 12.78±0.40 C | 3.34±0.08 D | 12.19±0.54 B |
| | $E_DM_D$ | 14.10±0.29 D | 2.22±0.25 C | 1.30±0.07 D | 0.99±0.02 D | 11.88±0.41 D | 2.15±0.45 E | 11.95±0.73 B |
| | $E_DM_R$ | 17.10±0.21 B | 3.06±0.13 B | 1.46±0.06 CD | 1.34±0.03 C | 14.04±0.09 B | 4.89±0.66 C | 12.21±0.45 B |
| | R | 19.78±0.47 A | 3.69±0.13 A | 2.28±0.01 B | 1.67±0.07 B | 16.09±0.36 A | 6.53±0.81 A | 13.25±0.38 A |
| | $E_RM_D$ | 19.17±0.26 A | 3.39±0.19 A | 2.16±0.14 B | 1.77±0.04 A | 15.77±0.22 A | 5.50±0.04 BC | 13.66±0.30 A |
| | $E_RM_R$ | 19.32±0.03 A | 3.60±0.12 A | 2.47±0.17 A | 1.84±0.04 A | 15.71±0.16 A | 6.22±0.28 AB | 13.10±0.29 A |

Values (means ± SD, $n = 3$) within the same column followed by different letters are significantly different at $P < 0.05$ according to Duncan's tests.

[a] Abbreviations: TOC - soil total organic carbon; $EOC_{333}$, $EOC_{167}$, and $EOC_{33.3}$ - different fractions of soil easily oxidized organic carbon; IOC - soil inert organic carbon; LFOC - soil light fraction organic carbon; HFOC - soil heavy fraction carbon. The treatment abbreviations are defined in Figure 1 and Table 1.

**Table S3**. The contents of nitrogen fraction, pH, and electrical conductivity in the different soils after incubation and planting

| | Treatments | TON[a] (g kg$^{-1}$) | C/N | $NH_4^+$-N (mg kg$^{-1}$) | $NO_3^-$-N (mg kg$^{-1}$) | pH | EC (mS cm$^{-1}$) |
|---|---|---|---|---|---|---|---|
| After incubation | D | 1.84±0.01 a | 9.82±0.27 c | 14.66±0.40 b | 592±42.14 a | 5.26±0.03 d | 0.88±0.01 b |
| | $E_DM_D$ | 1.88±0.00 a | 9.19±0.11 c | 24.31±0.07 a | 612±10.58 a | 5.42±0.02 c | 0.95±0.02 a |
| | $E_DM_R$ | 1.77±0.31 a | 10.05±1.68 bc | 23.61±0.16 a | 556±13.85 b | 5.79±0.01 b | 0.87±0.00 c |
| | R | 1.83±0.02 a | 11.84±0.13 a | 2.00±0.27 d | 0.00±0.00 c | 7.02±0.03 a | 0.40±0.00 f |
| | $E_RM_D$ | 1.81±0.01 a | 11.18±0.21 ab | 4.46±0.57 c | 1.51±1.24 c | 7.05±0.01 a | 0.43±0.00 e |
| | $E_RM_R$ | 1.76±0.01 a | 11.79±0.25 a | 1.50±0.86 d | 1.33±0.44 c | 7.04±0.01 a | 0.45±0.00 d |
| After planting | D | 1.82±0.13 A | 8.51±0.49 B | 19.83±0.27 B | 456±31.32 B | 5.31±0.03 F | 0.90±0.02 B |
| | $E_DM_D$ | 1.58±0.10 B | 8.93±0.77 B | 24.98±0.32 A | 494±16.09 A | 5.52±0.04 E | 0.94±0.03 A |
| | $E_DM_R$ | 1.87±0.07 A | 9.11±0.35 B | 26.23±2.11 A | 262±8.80 C | 5.82±0.01 D | 0.89±0.02 B |
| | R | 1.65±0.00 B | 11.96±0.32 A | 3.38±1.66 C | 3.53±0.15 D | 6.89±0.03 C | 0.41±0.01 C |
| | $E_RM_D$ | 1.73±0.04 AB | 11.07±0.23 A | 3.45±1.76 C | 4.00±0.08 D | 7.04±0.03 A | 0.43±0.00 C |
| | $E_RM_R$ | 1.61±0.10 B | 12.00±0.76 A | 2.40±0.90 C | 4.05±0.18 D | 6.98±0.01 B | 0.44±0.01 C |

Values (means ± SD, $n$ = 3) within the same column followed by different letters are significantly different at $P < 0.05$ according to Duncan's tests.

[a] Abbreviations: TON - soil total organic nitrogen; C/N - the ratio of soil total organic carbon to soil total organic nitrogen; EC - electrical conductivity. The treatment abbreviations are defined in Figure 1 and Table 1.

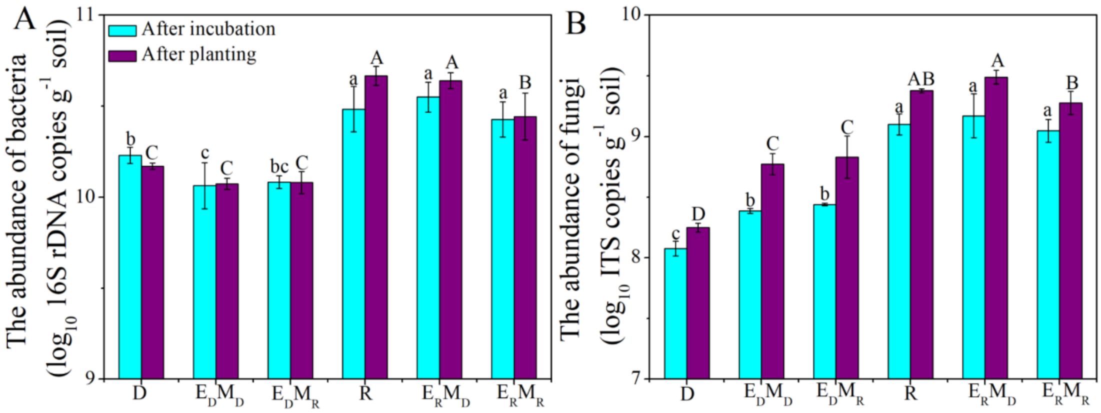


**Figure. S1** The abundances of bacteria (A) and fungi (B) in the different soils after incubation and planting. Bars with different letters represent significant differences according to Duncan's tests ($P < 0.05$) at after incubation (small letters) and planting (capital letters). Error bars represent SDs. The treatment abbreviations are defined in Figure 1 and Table 1.

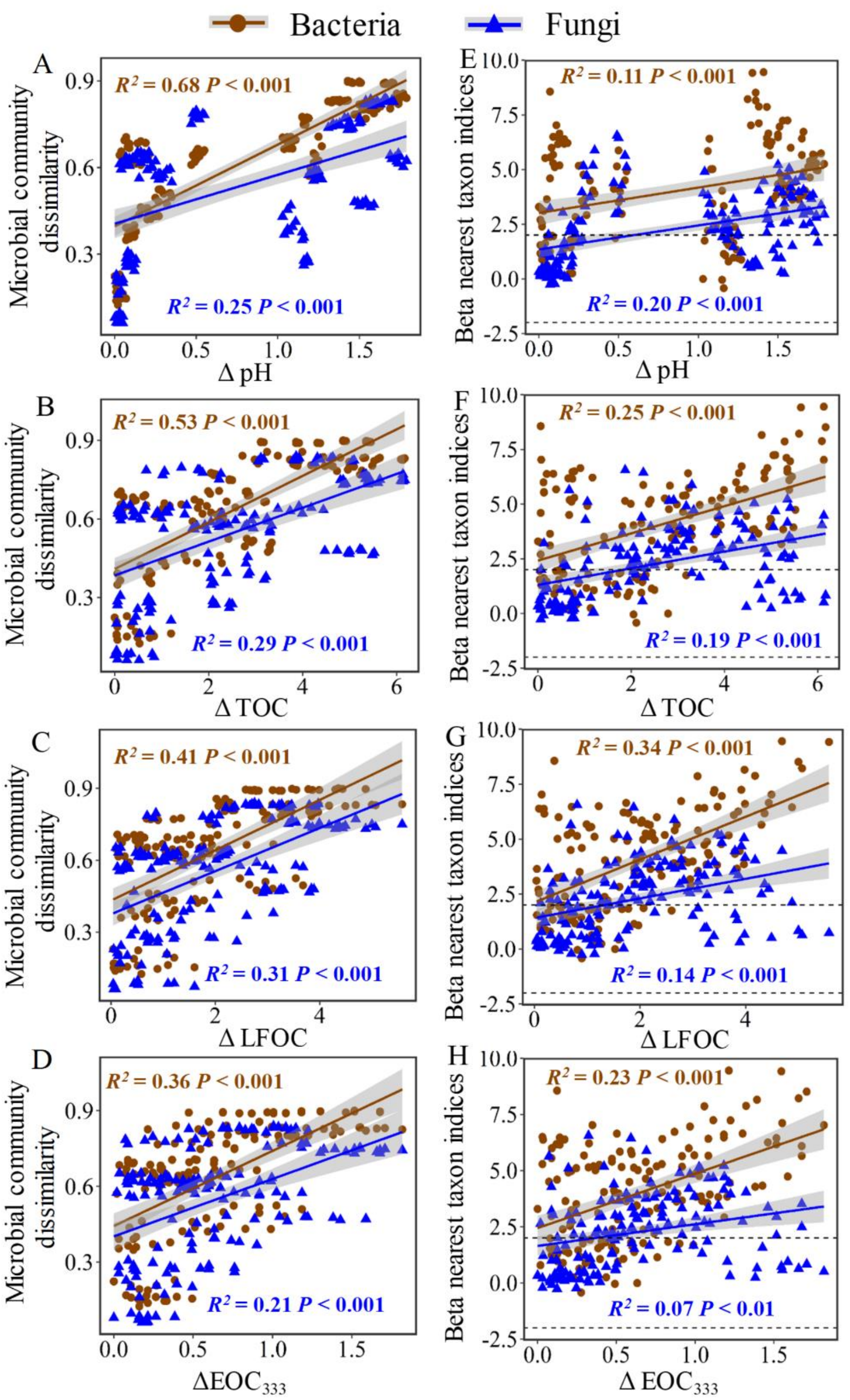


**Figure S2.** Relationships between the microbial community dissimilarities, pairwise beta nearest taxon indices (βNTI) and the differences in soil environmental factors. Dissimilarities in microbial community were calculated based on Bray–Curtis indices. Horizontal dashed lines indicate βNTI values of -2 and +2 in figure E-H. Δ pH, Δ TOC, Δ $EOC_{333}$, and Δ LFOC were calculated using Euclidean indices.

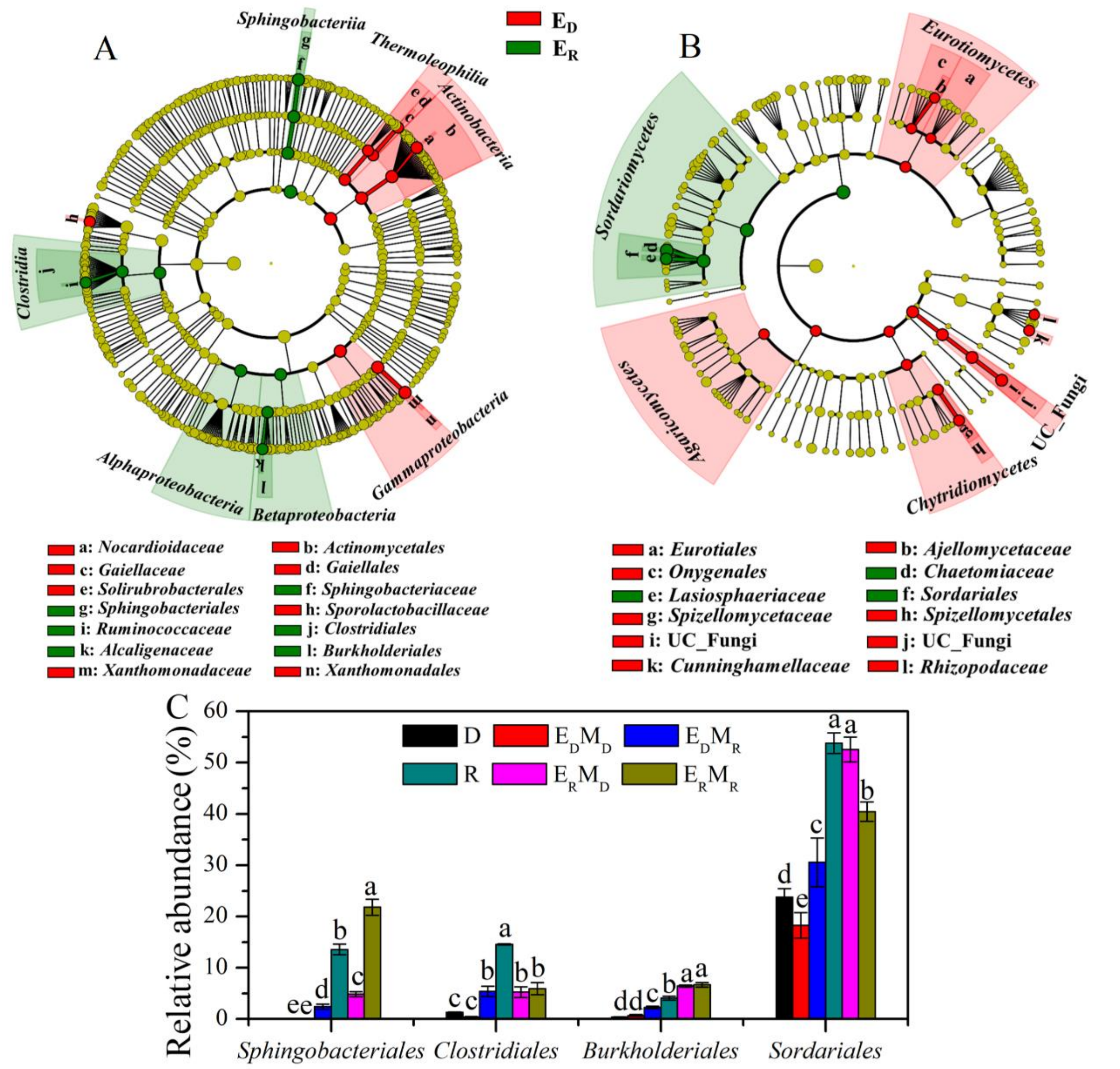


**Figure S3**. Differences in relative abundances of the bacterial (A) and fungal (B) taxa between $E_D$ (D, $E_DM_D$, and $E_DM_R$) and $E_R$ (R, $E_RM_D$, and $E_RM_R$) soils indicated by LDA Effect Size (LEfSe) analysis. Alpha value for the factorial Kruskal-Wallis test was 0.05, and LDA score threshold of bacteria and fungi was 4.0 and 3.5, respectively. The layers consisted by taxa from the innermost to the outermost represent kingdom, phylum, class, order, and family, respectively. The taxa significantly ($P < 0.05$) different in relative abundance are shown in red (importance in the $E_D$ soil) and green (importance in the $E_R$ soil), and the taxa not significantly different ($P > 0.05$) in relative abundance is shown in yellow. The size of circle indicates the normalized relative abundance of the taxa. C, the abundances of dominant microbial orders significantly varied between $E_D$

and $E_R$ soils. Error bars indicate SEs, and different letters indicate significant difference according to Duncan's test ($P < 0.05$). The treatment abbreviations are defined in Figure 1 and Table 1.

**The scripts for processing raw sequencing data**

**Analysis of bacterial 16S rDNA sequences**

**#Join paired-eds sequences with "fastq-join**

**1.** multiple_join_paired_ends.py -i Bacteria_raw_data/ -o Bacteria_joined_fastq_output --read1_indicator _R1 --read2_indicator _R2

**#Remove the primer sequences**

2. multiple_extract_barcodes.py -i Bacteria_joined_fastq_output/ -o Bacteria_no_primers -p Parameter/multiple_extract_barcodes_parameters.txt --include_input_dir_path --remove_filepath_in_name

**#Quality filtering**

3. multiple_split_libraries_fastq.py -i Bacteria_no_primers/ -o Bacteria_split_fastq_output --demultiplexing_method sampleid_by_file --include_input_dir_path --remove_filepath_in_name -p Parameter/multiple_split_parameters.txt

**###Make OTU tables & pick OTUs based on sequences similarity within the reads using an open reference picking strategy (Greengene13_8)**

**4.** pick_open_reference_otus.py -i Bacteria_split_fastq_output/seqs.fna -r Database/97_otus.fasta -o Bacteria_uclust_otus/ -p pick16sotu_inputpara.txt -s 0.1 --prefilter_percent_id 0.0 -m uclust

**#Identify the chimeric OTUs**

5. parallel_identify_chimeric_seqs.py -i bacteria_uclust_otus/pynast_aligned_seqs/rep_set_aligned.fasta -a core_set_aligned.fasta.imputed -o bacteria_chimeric_otus.txt -O 6

**#Filter chimeric OTUs from OTU tables**

6. filter_otus_from_otu_table.py -i bacteria_uclust_otus/otu_table_mc2_w_tax_no_pynast_failures.biom -o otu_table_mc2_w_tax_no_pynast_failures_no_chimera.biom -e bacteria_chimeric_otus.txt

**#Convert BIOM format to txt format to identify the Chloroplast and Mitochondria OTUs (bacteria_chloroplast_mitochondria_otus.txt)**

7. biom convert -i otu_table_mc2_w_tax_no_pynast_failures_no_chimera.biom -o otu_table_mc2_w_tax_no_pynast_failures_no_chimera.txt --to-tsv --header-key taxonomy

**#Filter the chloroplast and mitochondria OTUs from OTU tables**

8. filter_otus_from_otu_table.py -i otu_table_mc2_w_tax_no_pynast_failures_no_chimera.biom -o otu_table_mc2_w_tax_no_pynast_failures_no_chimera_chloroplast_mitochondria.biom -e bacteria_chloroplast_mitochondria_otus.txt

**#Rename the OTU tables**

9. cp otu_table_mc2_w_tax_no_pynast_failures_no_chimera_chloroplast_mitochondria.biom bacteria_final_otu_table.biom

**#Count the number of seqs and OTUs**

10. biom summarize-table -i bacteria_final_otu_table.biom -o bacteria_table_summary.txt ##seqs

biom summarize-table -i bacteria_final_otu_table.biom -o bacteria_table_summary_otus.txt --qualitative ##otus

**###Make the phylogenetic tree & #Filter the chimeric sequences from fasta file**

**11.** filter_fasta.py -f bacteria_uclust_otus/pynast_aligned_seqs/rep_set_aligned.fasta -o bacteria_rep_set_aligned_no_chimera.fasta -s bacteria_chimeric_otus.txt -n

**#Filter the chloroplast and mitochondria sequences from the above fasta file**

**12.** filter_fasta.py -f bacteria_rep_set_aligned_no_chimera.fasta -o bacteria_rep_set_aligned_no_chimera_chloroplast_mitochondria_fasta -s bacteria_chloroplast_mitochondria_otus.txt -n

**#Filter sequence alignment by removing highly variable regions**

**13.** filter_alignment.py -i bacteria_rep_set_aligned_no_chimera_chloroplast_mitochondria_fasta -o bacteria_rep_set_aligned_no_chimera_chloroplast_mitochondria_pfiltered.fasta

**#make phylogenetic tree**

**14.** make_phylogeny.py -i bacteria_rep_set_aligned_no_chimera_chloroplast_mitochondria_pfiltered.fasta/bacteria_rep_set_aligned_no_chimera_chloroplast_mitochondria_fasta_pfiltered.fasta -o bacteria_rep_set_aligned_no_chimera_chloroplast_mitochondria.tre -t fasttree

**#Rename the phylogenetic tree**

**15.** cp bacteria_rep_set_aligned_no_chimera_chloroplast_mitochondria.tre bacteria_final_rep_set.tre

**#Rarefy the OTU table and then convert to txt format**

16. single_rarefaction.py -i bacteria_final_otu_table.biom -o bacteria_final_otu_table_even26000.biom -d 26000

17. biom convert -i bacteria_final_otu_table_even26000.biom -o bacteria_final_otu_table_even26000.txt --to-tsv --header-key taxonomy

**#Summarize taxonomy composition based on description and treatment**

18. summarize_taxa_through_plots.py -i bacteria_final_otu_table_even26000.biom -o 16S_taxonomy/Description -m 16s_map.txt -c Description

summarize_taxa_through_plots.py -i bacteria_final_otu_table_even26000.biom -o 16S_taxonomy/Treatment -m 16s_map.txt -c Treatment

**#Aplha diversity analysis**

19. alpha_diversity.py -i bacteria_final_otu_table_even26000.biom -m ace,chao1,equitability,goods_coverage,observed_species,shannon,simpson,PD_whole_tree -t bacteria_final_rep_set.tre -o bacteria_alpha_diversity_index.txt

**Analysis of fungal ITS sequences**

**#Join paired-eds sequences with "fastq-join"**

1. multiple_join_paired_ends.py -i Fungi_raw_data/ -o Fungi_joined_fastq_output --read1_indicator _R1 --read2_indicator _R2

**#Remove the primer sequences**

2. multiple_extract_barcodes.py -i Fungi_joined_fastq_output/ -o Fungi_no_primers -p Parameter/multiple_extract_barcodes_parameters.txt --include_input_dir_path --remove_filepath_in_name

**#Trunclen 200bp in each reads using Usearch , and all of the final output fastq files are put in a Usearch folder (1-18 samples)**

3. Usearch -fastq_filter Fungi_no_primers/1_R1/reads.fastq -fastqout 1_R1_200.fastq -fastq_trunclen 200

**#Quality filtering**

4. multiple_split_libraries_fastq.py -i Usearch/ -o Fungi_split_fastq_output --demultiplexing_method sampleid_by_file --include_input_dir_path --remove_filepath_in_name -p Parameter/multiple_split_parameters.txt

**#Pick OTUs based on sequences similarity within the reads using an open reference picking strategy(UNITE database)**

5. pick_open_reference_otus.py -i Fungi_split_fastq_output/seqs.fna -r sh_qiime_release_01.08.2015/sh_refs_qiime_ver7_97_01.08.2015.fasta -o Fungi_uclust_otus/ -p pickitsotu_inputpara2.txt -s 0.1 --prefilter_percent_id 0.0 --suppress_align_and_tree

**#Identify the chimeric OTUs**

6. Usearch -uchime2_ref rep_set.fna -db sh_qiime_release_01.08.2015/sh_refs_qiime_ver7_97_01.08.2015.fasta -strand plus -mode sensitive -uchimeout chimeras.txt

**#Convert BIOM format to txt format to identify the chimera otus (Fungi_chimera_otu.txt)**

7. biom convert -i Fungi_uclust_otus/otu_table_mc2_w_tax.biom -o Fungi_otu_table_mc2_w_tax.txt --to-tsv --header-key taxonomy

**#Filter chimeric OTUs from OTU tables**

8. filter_otus_from_otu_table.py -i Fungi_otu_table_mc2_w_tax.biom -o _Fungi_nochimeras.biom -e Fungi_chimera.txt

**#Count the number of seqs and OTUs**

9. biom summarize-table -i Fungi_nochimeras.biom -o its_counts_summary.txt ##seqs

   biom summarize-table -i Fungi_nochimeras.biom -o its_otus_summary.txt --qualitative ##otus

**#Rename the OTU tables**

10. cp Fungi_nochimeras.biom Fungi_final_otu_table.biom

**#Rarefy the OTU table and then convert to txt format**

11. single_rarefaction.py -i Fungi_final_otu_table.biom -o Fungi_final_otu_table_even45000.biom -d 45000

    biom convert -i Fungi_final_otu_table_even45000.biom -o Fungi_final_otu_table_even45000.txt --to-tsv --header-key taxonomy

**#Summarize taxonomy composition based on description and treatment**

12. summarize_taxa_through_plots.py -i Fungi_final_otu_table_even45000.biom -o its_taxonomy/Description -m map.txt -c Description

    summarize_taxa_through_plots.py -i Fungi_final_otu_table_even45000.biom -o its_taxonomy/Treatment -m map.txt -c Treatment

**#Alpha diversity**

13. alpha_diversity.py -i Fungi_final_otu_table_even45000.biom -m ace,chao1,equitability,goods_coverage,observed_species,shannon,simpson -o its_alpha_diversity.txt
